# Bacteria-based cascade *in situ* near-infrared nano-optogenetically induced photothermal tumor therapy

**DOI:** 10.7150/thno.98097

**Published:** 2024-08-12

**Authors:** Xiuwen Hu, Jiawen Chen, Yuzhi Qiu, Sihan Chen, Yidi Liu, Xi Yu, Yunting Liu, Xiangliang Yang, Yan Zhang, Yanhong Zhu

**Affiliations:** National Engineering Research Center for Nanomedicine, College of Life Science and Technology, Huazhong University of Science and Technology, 1037 Luoyu Road, Wuhan 430074, P. R. China.

**Keywords:** NIR-optogenetics, bacteria, α-hemolysin, *in situ* photothermal agents, tumor therapy

## Abstract

**Rationale:** Optogenetically engineered facultative anaerobic bacteria exhibit a favorable tendency to colonize at solid tumor sites and spatiotemporally-programmable therapeutics release abilities, attracting extensive attention in precision tumor therapy. However, their therapeutic efficacy is moderate. Conventional photothermal agents with high tumor ablation capabilities exhibit low tumor targeting efficiency, resulting in significant off-target side effects. The combination of optogenetics and photothermal therapy may offer both tumor-targeting and excellent tumor-elimination capabilities, which unfortunately has rarely been investigated. Herein, we construct a bacteria-based cascade near-infrared optogentical-photothermal system (EcN_αHL_-UCNPs) for enhanced tumor therapy.

**Methods:** EcN_αHL_-UCNPs consists of an optogenetically engineered Escherichia coli Nissle 1917 (EcN) conjugated with lanthanide-doped upconversion nanoparticles (UCNPs), which are capable of locally secreting α-hemolysin (αHL), a pore-forming protein, in responsive to NIR irradiation. Anti-tumor effects of EcN_αHL_-UCNPs were determined in both H22 and 4T1 tumors.

**Results:** The αHL not only eliminates tumor cells, but more importantly disrupts endothelium to form thrombosis as an *in situ* photothermal agent in tumors. The *in situ* formed thrombosis significantly potentiates the photothermic ablation of H22 tumors upon subsequent NIR light irradiation. Besides, αHL secreted by EcN_αHL_-UCNPs under NIR light irradiation not only inhibits 4T1 tumor growth, but also suppresses metastasis of 4T1 tumor via inducing the immune response.

**Conclusion:** Our studies highlight bacteria-based cascade optogenetical-photothermal system for precise and effective tumor therapy.

## Introduction

The utilization of anaerobic or facultative anaerobic bacteria in cancer therapy dates back to more than a century ago, due to their tendency to colonize in the hypoxic tumor sites 1, 2. Recent progress in synthetic biology has facilitated to engineer bacteria with therapeutic genes as living drug-producing factories for improved therapeutic efficacy 3. However, the therapeutic genes in most of these engineered bacteria are constitutive expressed, suffering from off-target toxicity and unsatisfactory therapeutic outcomes that hamper their clinical applications 4, 5.

Optogenetics with optical-responsive gene switches enables light-controllable cellular behavior manipulation with high spatial and temporal resolution 6, 7, thus opening up exciting opportunities for traceless, remotely-controlled precision medicine 8. Existing optogenetic systems perform well in selected contexts but are often limited by low tissue penetration. Therefore, intrusive optic-fiber implantation has been commonly utilized in optogenetic manipulation 9-11. To penetrate tissue deeply with minimal invasiveness, we and others have developed near-infrared (NIR) light responsive optogenetic systems, in which lanthanide-doped upconversion nanoparticles (UCNPs) were used to convert NIR light to blue light to indirectly activate the optogenetic gene expression in the engineered bacteria for tumor therapy 12-14. These NIR light-responsive optogenetic systems inhibited tumor growth to a large extent, but unfortunately, they couldn't completely ablate tumors.

Photothermal therapy (PTT) commonly utilizes photothermal agents (PTAs) to convert light energy into heat and has been extensively utilized in tumor treatment 15. To date, conventional PTAs including dye molecules, photosensitizer polymers, noble metallic nanomaterials, carbon-based nanomaterials, and semiconductor nanoparticles, have been fabricated with variable anti-tumor therapeutic efficacy 16. However, they may cause severe damage to normal tissue due to the limited targeting abilities via i.v. injection. Therefore, *in situ* formed PTAs in tumors are highly desirable. Vasodilator inflammatory factors, such as, platelet-activating factor (PAF), leukotriene C4 (LTC4), histamine (His), and bradykinin (BK), can disrupt tumor vasculature to induce blood influx and platelet aggregation to form thrombosis in the extravascular area within tumors 17, 18. Such tumor thrombosis exhibits high photothermal conversion capability and can act as PTAs 18, whereas unfortunately, spatiotemporally controllable formation of thrombosis has been neglected in cancer treatments. Moreover, to the best of our knowledge, optogenetics induced tumor *in situ* formed thrombosis as PTA has rarely been reported.

**Figure 1:**
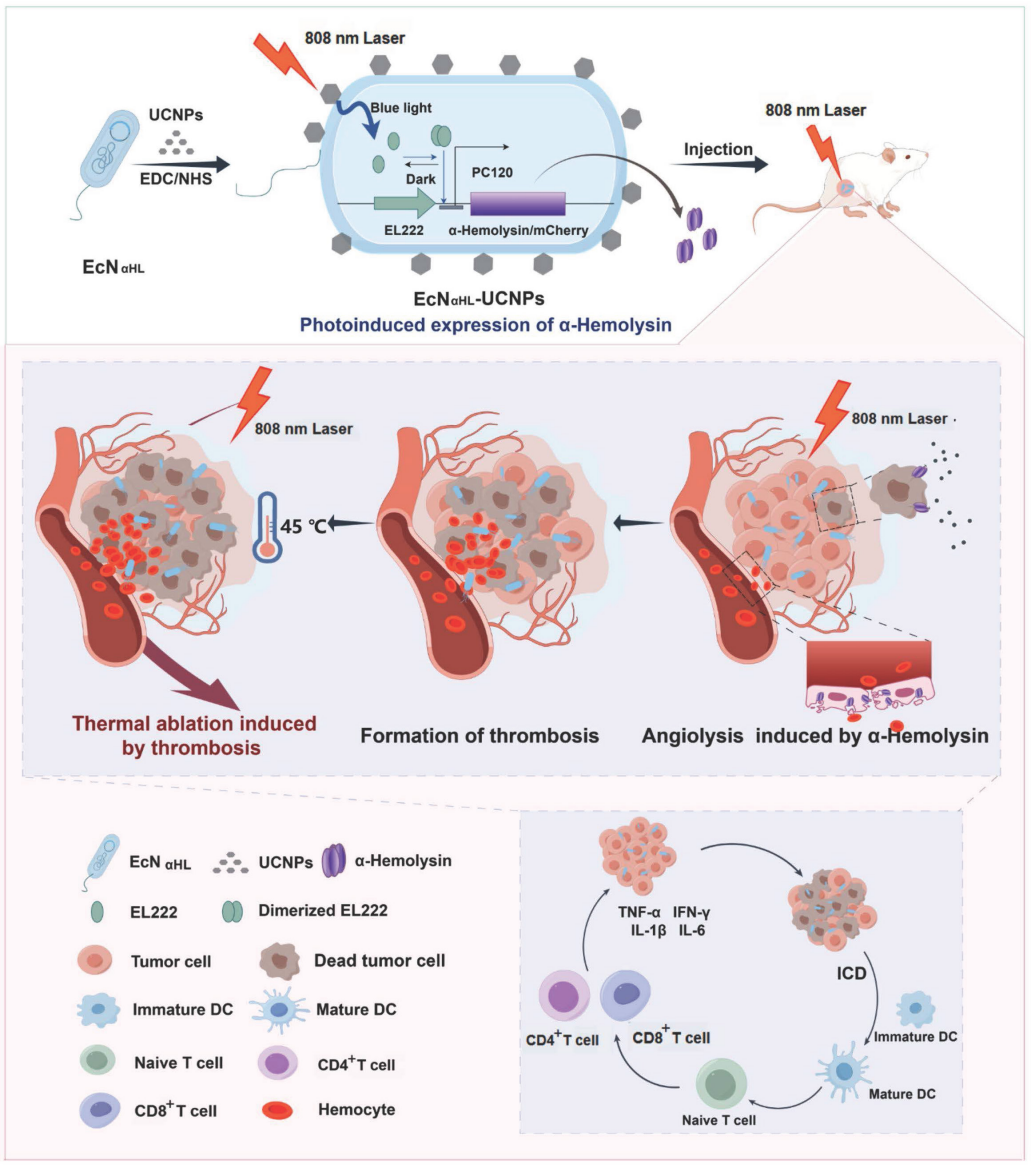
Schematic illustration of the construction of EcN_αHL_-UCNPs and its antitumor immune response via NIR optogenetically releasing αHL and thrombosis formed subsequently for synergistic photothermal therapy.

In this study, a bacteria-based near-infrared optogenetically *in situ* induced photothermal cancer therapy is reported. Escherichia coli Nissle 1917 (EcN), one of the probiotics 19, is genetically engineered to express α-hemolysin (αHL), a pore-forming protein 20, upon blue light irradiation (Fig.1). The engineered EcN is further conjugated with UCNPs, which converts NIR light to blue light to induce αHL expression and secretion from the engineered EcN. αHL in tumors not only elicits tumor cell death, but more importantly disrupts tumor endothelium, resulting in an influx of blood cells into the extravascular matrix within tumors where thrombus occurs 21. Such thrombus with strong NIR absorbance enables accurate photothermal therapy to completely inhibit H22 tumor growth with minimal off-target toxicity that is rarely seen in purely genetically engineered bacterial systems. Moreover, the αHL secretion triggers the immune response and inhibits 4T1 tumor metastasis. Thus, the bacteria-based optogenetically induced *in situ* PTT holds great promise in precision cancer therapy.

## Results and Discussion

### Preparation of EcN_αHL_-UCNPs and its characterization

**Figure 2:**
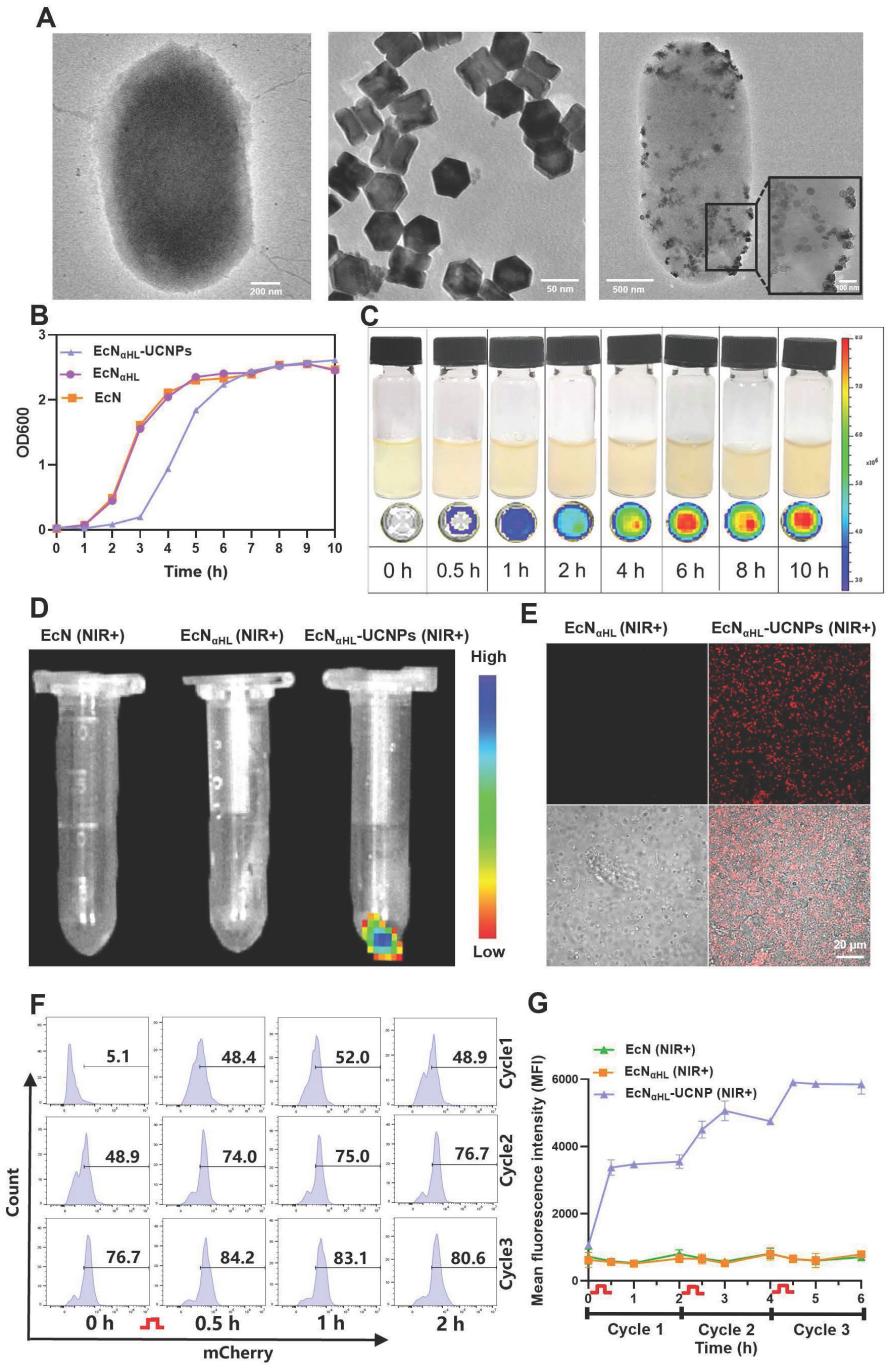
**Characteristics of EcN_αHL_-UCNP**. **(A)** TEM images of EcN_αHL_, UCNPs and EcN_αHL_-UCNP. **(B)** The OD600 values of EcN, EcN_αHL_ and EcN_αHL_-UCNP at different time points. **(C)** Fluorescence images of EcN_αHL_ induced by blue light in liquid culture medium at different time points. **(D)** Fluorescence images and **(E)** confocal images of EcN, EcN_αHL_ and EcN_αHL_-UCNP irradiated by NIR (1 W cm^-2^, 30 min), Scale bar: 20 μm. **(F-G)** The ratio of EcN_αHL_-UCNP (+) and quantitative analysis of mCherry positive EcN_αHL_ (n = 3). 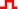 means NIR irradiation (1 W cm^-2^, 30 min).

The genetic sequences of blue light-responsive EL222 sensor protein and αHL were introduced into the plasmid pET20b to construct the blue light-activatable secretory αHL expressing system in EcN, while mCherry was used as a fluorescent reporter. In light of the limited penetration of blue light, UCNPs with the ability to convert deeper tissue-penetrating 808 nm NIR light to blue light 22, 23, were conjugated to EcN_αHL_ via EDC/NHS chemistry. UCNPs were synthesized according to our previous report 24. As shown in Figure 2A, UCNPs (TEM image) were monodispersed. An average diameter of UCNPs determined to be 66.3 ± 8.2 nm by DLS. After modification by L-Ala, UCNPs were positively charged with zeta potential 27.70 ± 1.45 mV. The size of EcN_αHL_ was 1642.33 ± 69.29 nm, and zeta potential was -21.37 ± 0.91 mV. After conjugation of UCNPs, the size of EcN_αHL_-UCNPs was achieved to 2588.00 ± 75.94 nm, while zeta potential was 0.07 ± 0.07 mV (Figure S2A-B). Under irradiation of 808 nm NIR, EcN_αHL_-UCNPs and UCNPs had similar emission peaks, while EcN_αHL_ presented no significant peak (Figure S2C).

OD600 value is generally utilized to determine the content of bacteria in medium 25. As shown in Figure 2B, EcN_αHL_-UCNPs had a similar growth curve to EcN_αHL_. UCNPs had no significant effects on the colony formation of EcN_αHL_ (Figure S3). The growth rate of EcN_αHL_-UCNPs in the early 7 h was slower than that of EcN_αHL_, thereafter EcN_αHL_-UCNPs achieved to a stable stage similar to EcN and EcN_αHL_. Upon blue light irradiation, the fluorescence intensity of EcN_αHL_ was increased with the extension of irradiation time (Figure 2C). EcN_αHL_-UCNPs can emit mCherry fluorescence under confocal microscope when irradiated with NIR light, and similar results were observed by IVIS Lumina XR system (Figure 2D-E). Moreover, western blot analyses showed that a significant amount of mCherry protein in EcN_αHL_ was induced by blue light, EcN_αHL_-UCNPs induced by NIR and the supernatant in the EcN_αHL_-UCNPs (NIR+) group (Figure S4). The mCherry signal increased with the enhanced illumination power intensity (Figure S5).

To further investigate the NIR irradiation-dependent expression of αHL in EcN_αHL_-UCNPs, fluorescence intensity was measured by flow cytometry every 2 h after NIR illumination for 30 min, 3 cycles (Figure 2F-G). Fluorescence intensity was found to be increased after each illumination. Therefore, the expression of αHL in EcN_αHL_-UCNPs can be regulated by NIR irradiation.

### NIR light-activatable EcN_αHL_-UCNPs secrete αHL to induce tumor cell death

**Figure 3:**
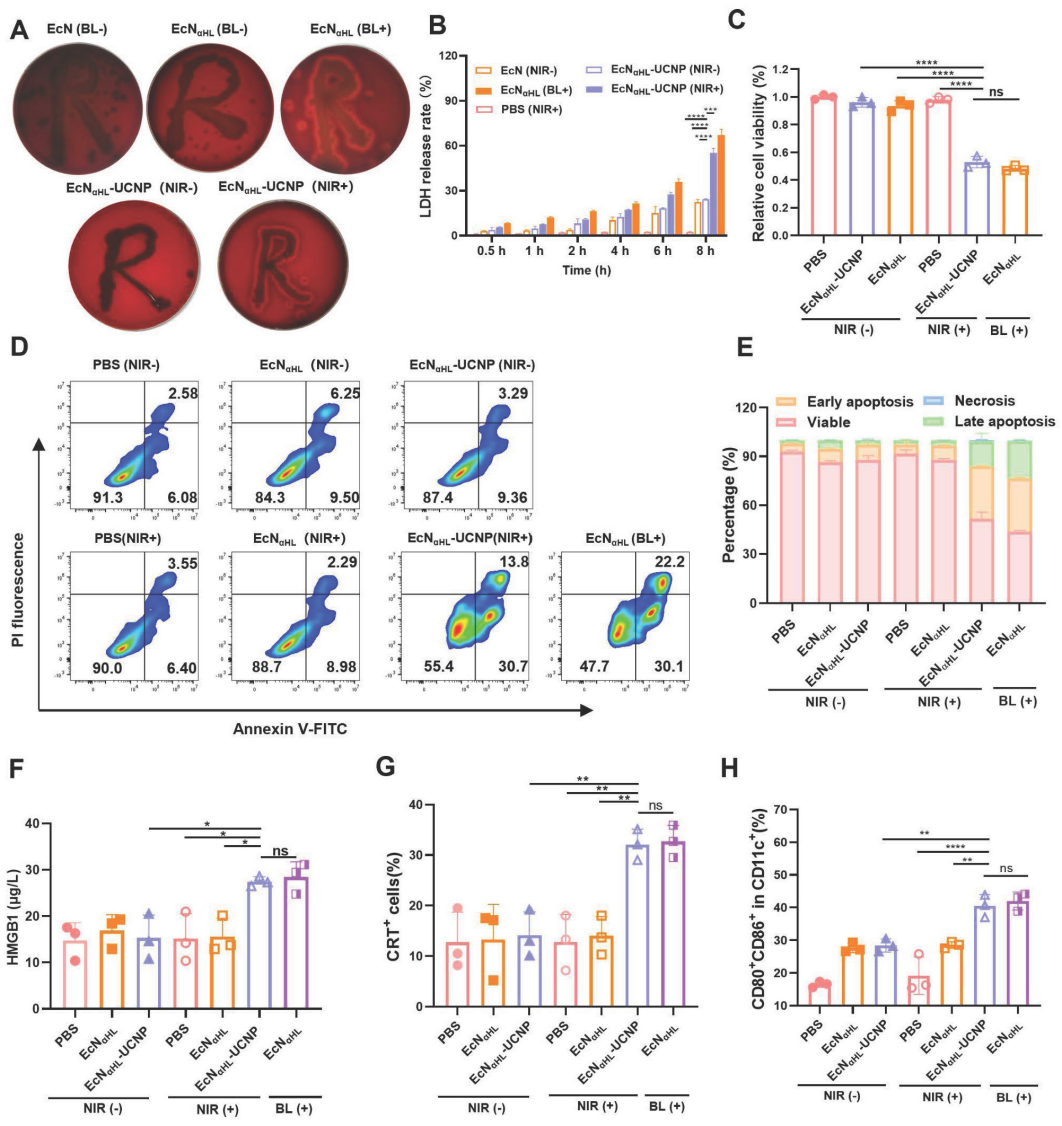
***In vitro* evaluation of antitumor efficacy. (A)** Images of the hemolysis phenomenon of EcN, EcN_αHL_ and EcNαHL-UCNPs with or without irradiation on blood agar plates. **(B)** At different time points, LDH release rate of 4T1 cells treated by EcN_αHL_-UCNPs irradiated with NIR (1 W cm^-2^, 2 h). **(C)** The relative cell viability of 4T1 cells treated by EcN_αHL_-UCNPs irradiated with or without NIR. **(D)** Apoptosis of 4T1 cells induced by different treatments and proportion of different cell statuses **(E)**. ICD in 4T1 cells induced by EcN_αHL_-UCNPs irradiated with NIR. HMGB1 in supernatants measured by ELISA kit **(F)** and fluorescence intensity of CRT detected by flowcytometry **(G)**. **(H)** Activation of BMDCs *in vitro*. (*p < 0.05, **p < 0.01, ***p < 0.001, and ****p < 0.0001, ns: no significant difference).

The α-hemolysin (αHL) protein is a pore-forming toxin secreted by Staphylococcus aureus, that has the most distinct hemolytic behavior 21. In this study, blood agar plate was used to test the αHL expression and secretion by EcN_αHL_. As shown in Figure 3A, EcN_αHL_ and EcN_αHL_-UCNPs can induce hemolysis and present a hemolytic ring after irradiation. Lactate dehydrogenase (LDH) as one content in most living cells, will be released to the extracellular side when cell membrane integrality is disrupted. After incubation with EcN_αHL_-UCNPs irradiated with NIR for 2 h, 4T1 cells released LDH and the level of LDH in the supernatant increased over time. After eight h, the relative release rate achieved 55%, whereas those of EcN_αHL_-UCNPs and EcN_αHL_ were 22% and 24% without illumination, respectively (Figure 3B), which might be attributed to pore-forming on cell membrane by insertion of αHL. To measure the cytotoxicity of irradiated EcN_αHL_-UCNPs, cell viability was determined by the MTT assay. After incubation with the supernatants of EcN_αHL_-UCNPs and EcN_αHL_ illuminated with NIR or blue light for 4 h, viability of 4T1 cells was decreased to 53% and 48%, respectively. EcN_αHL_-UCNPs and EcN_αHL_ groups without illumination had the similar cell viabilities to that of PBS group (Figure 3C). Similar results were observed in H22 cell viability measured by Cell Counting Kit 8 (CCK8) after different treatments (Figure S6). These results demonstrated that EcN_αHL_-UCNPs can kill tumor cells under NIR illumination.

We further investigated the effects of NIR-illuminated EcN_αHL_-UCNPs on apoptosis of 4T1 (Figure 3D-E). Early apoptosis accounted for 30.7% and advanced apoptosis accounted for 13.8% in the EcN_αHL_-UCNPs (NIR+) groups, whereas 6.40% for early apoptosis and 3.55% for advanced apoptosis in the PBS group. Cells in EcN_αHL_-UCNPs (NIR-) and EcN_αHL_ (NIR-) groups had similar viabilities.

### Immunogenic cell death of tumor cells triggered by EcN_αHL_-UCNPs (NIR+) *in vitro*

Immunogenic cell death (ICD) is often accompanied by the release of immunostimulatory damage-associated molecular patterns (DAMPs) such as surface-exposed calreticulin (CRT) and high extracellular secretion of mobility histone B1 (HMGB1) 26, 27. In this study, EcN_αHL_-UCNPs (NIR+) could increase the level of HMGB1, which was 1.9-fold higher than that of PBS (Figure 3F). The flow cytometry results showed that EcN_αHL_-UCNPs (NIR+) could promote the CRT expression in 4T1 cells (Figure 3G).

DAMPs can be presented by antigen presenting cells (APCs) to induce immune response. We examined dendritic cells (DC) maturation (CD11c^+^ CD80^+^ CD86^+^) induced by ICD. Bone marrow derived dendritic cells (BMDCs) from BALB/c mice treated with cytokines IL-4 and GM-CSF, were incubated with the supernatants of EcN_αHL_-UCNPs with or without NIR irradiation, respectively, and then analyzed by flow cytometry. The results showed that the percentage of mature DCs in the EcN_αHL_-UCNPs (NIR+) accounted for 40.7%, which was significantly higher than those of the other groups without illumination (Figure 3H and Figure S7). Taken together, EcN_αHL_-UCNPs (NIR+) induce the cytotoxicity and ICD of 4T1 tumor cells to promote DCs maturation.

### Biodistribution of EcN_αHL_-UCNPs in tumor-bearing mice

Due to the targeting ability of EcN to hypoxia microenvironment, we deduce that EcN_αHL_-UCNPs have the tendency to colonize in solid tumors as well. Here, we examined the targeting ability of EcN_αHL_-UCNPs in the subcutaneous 4T1 tumor murine model. 1×10^6^ CFU IR780-labeled EcN_αHL_ or EcN_αHL_-UCNPs were intravenously injected into each mouse when tumor volume reached 100-150 mm^3^, while the same volume of PBS used as a negative control. The animal *in vivo* imaging system (IVIS) results showed that the strong fluorescence intensity maintained at 24-72 h at tumor sites after intravenous injection of EcN_αHL_-UCNPs (Figure S8). The distribution of EcN_αHL_-UCNPs in main organs and tumors was evaluated. The hearts, livers, spleens, lungs, kidneys, and tumors of mice were respectively collected at different time points after injection of EcN_αHL_-UCNPs suspensions. Yb, Nd and Y of UCNPs in tumor tissues were far higher than those in other organs after injection for 72 h through ICP-OES analysis (Figure S9). EcN_αHL_-UCNPs in the homogenates of these tissues were cultured and quantified. As seen in Figure S10, compared to main organs, bacterial counts were continuously increased in tumors with increasing time after administration, and maintained high bacterial level even on day 7.

### Therapeutic effects of NIR light-activatable EcN_αHL_-UCNPs on subcutaneous H22 tumor-bearing mice

**Figure 4:**
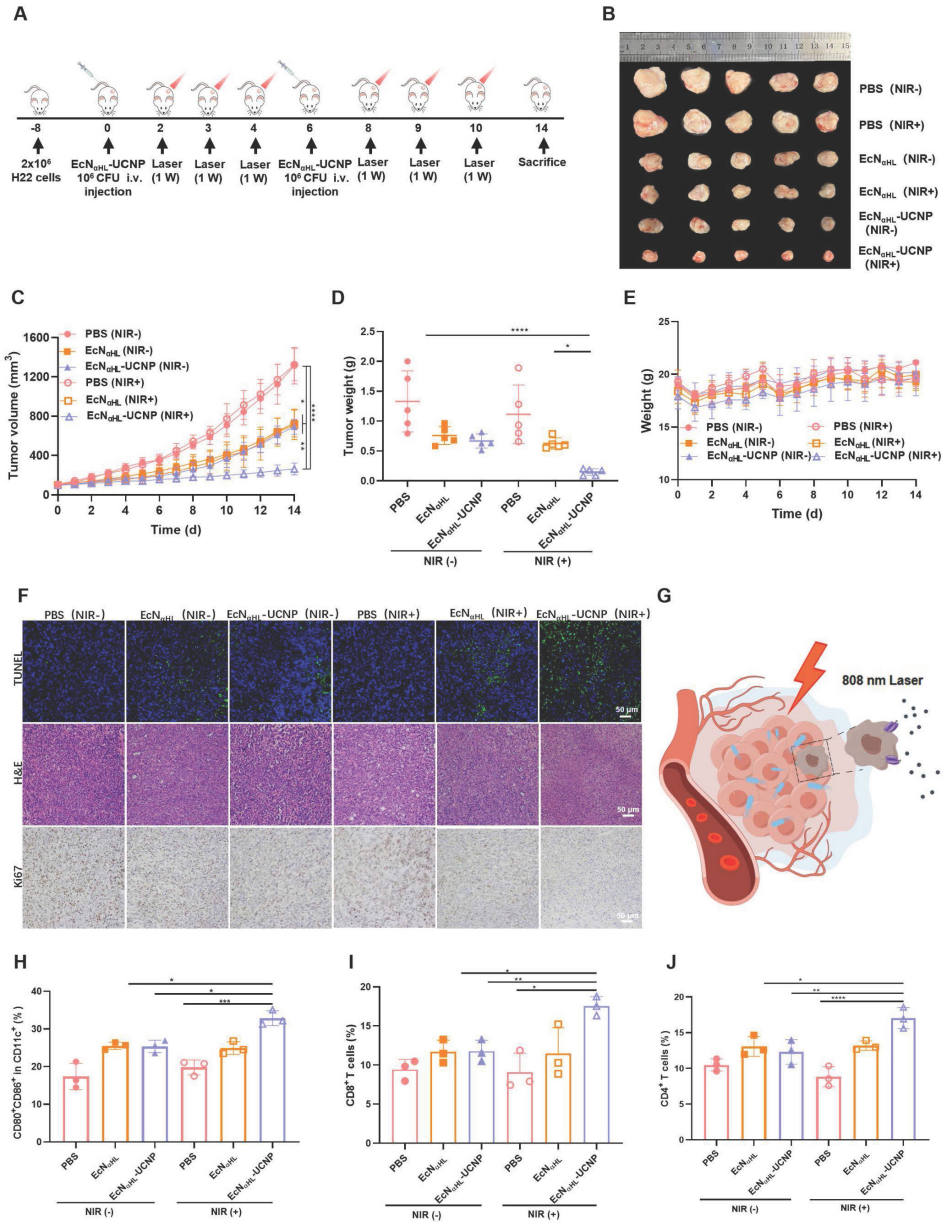
**Therapeutic effects of EcN_αHL_-UCNPs (NIR+) on subcutaneous H22 tumor-bearing mice. (A)** Experimental design for evaluating the effects of EcN_αHL_-UCNPs on subcutaneous H22 tumor-bearing mice. Tumor cells were inoculated on day -8 and 1×10^6^ CFU EcN_αHL_ or EcN_αHL_-UCNPs in 100 μL PBS were intravenously injected on day 0 and 6 respectively upon the tumor volume reaching 100-150 mm^3^ . Tumors were irradiated with an NIR laser (808 nm, 1 W cm^-2^, 10 min) on day 2, 3, 4, 8, 9 and 10, respectively. **(B)** H22 tumor images after different treatments **(C)** H22 tumor volume after different treatments. **(D)** Tumor weight and **(E)** Body weight. **(F)** H&E staining, Ki67 and TUNEL immunohistochemical staining images of tumors in different groups. **(G)** Scheme of EcN_αHL_-UCNPs (NIR+) killing tumor cells via NIR optogenetically releasing αHL. Scale bar: 50 μm. **(H)** Percentages of CD80^+^ CD86^+^ cells in CD11c^+^ cells in draining lymph nodes. **(I, J)** Percentages of CD3^+^ CD8^+^ T cells and CD3^+^ CD4^+^ T cells in tumor tissues. (*p < 0.05, **p < 0.01, ***p < 0.001, and ****p < 0.0001, ns: no significant difference).

To evaluate the efficacy of EcN_αHL_-UCNPs (NIR+) in the relative “hot” tumor, H22 tumor model was utilized (Figure 4A). Six groups were divided as follows: PBS (NIR-), PBS (NIR+), EcN_αHL_ (NIR-), EcN_αHL_ (NIR+), EcN_αHL_-UCNP (NIR-) and EcN_αHL_-UCN (NIR+). Tumor inhibition rate was 80.12% in EcN_αHL_-UCNP (NIR+) group (Figure 4B-D), higher than that in other groups. Tumor growth in EcN_αHL_ (NIR-), EcN_αHL_ (NIR+) and EcN_αHL_-UCNP (NIR-) groups were also inhibited, which may be attributed to the poor immunogenicity of EcN_αHL_ for tumor suppression. Meanwhile, all treated mice showed no significant differences in body weights (Figure 4E).

In addition, more apoptosis was shown in tumor tissues after treatment with EcN_αHL_-UCNPs (NIR+) than those in all other treatment groups through TUNEL staining and hematoxylin and eosin (H&E) staining, while less proliferation was presented in EcN_αHL_-UCNPs (NIR+) through immunohistochemical staining of Ki67 (Figure 4F). Meanwhile, in the heart, liver, spleen, lung, and kidney tissues of mice after different treatments, no obvious pathological changes were found through H&E staining (Figure S11). Taken together, these results demonstrated that EcN_αHL_-UCNPs (NIR+) presented significant tumor inhibitory efficacy.

The tumor drained lymph nodes were collected for analysis of DC activation on day 3 after first treatment. The CD80^+^CD86^+^ DCs in lymph nodes in EcN_αHL_-UCNPs (NIR+) group were 1.30-fold and 1.29-fold higher than those of EcN_αHL_-UCNPs without NIR light illumination group and EcN_αHL_ group, respectively (Figure 4H and Figure S12A). The proportion of CD8^+^ T cells in CD3^+^ T cells in tumor tissues after treatment with EcN_αHL_-UCNPs (NIR+) was higher than that in other groups, indicating that EcN_αHL_-UCNPs (NIR+) could promote the infiltration of CD8^+^ T cells into tumor tissues (Figure 4I and Figure S12B). Moreover, the proportion of CD4^+^ T cells in tumor tissues of H22 mice in the EcN_αHL_-UCNPs (NIR+) group was significantly higher than that in other groups (Figure 4J). The proportion of CD80^+^CD86^+^ DCs in tumor tissues treated with EcN_αHL_-UCNPs (NIR+) was also higher than that in other groups (Figure S13).

### Optogenetics-based photothermal therapy effects induced by EcN_αHL_-UCNPs (NIR+) on subcutaneous H22 tumor-bearing mice

α-hemolysin(αHL), as a water-soluble monomer that assembles into a heptamer to form a transmembrane pore on a target membrane, can induce cell death and lysis 28. After colonization of EcN_αHL_-UCNPs in tumor, αHL would be secreted into tumor microenvironments upon NIR illumination, thereby disrupting tumor cells and tumor vascular endothelial cells to induce hemolysis in tumor tissues. Hemolysis induces a significant increase of tumor necrosis factor-α both in blood and in vascular tissues 29. Conversely, TNF-α disrupts endothelial cell functions, resulting in endothelial procoagulant activity which may play a role in disseminated intravascular coagulation 17, 30. The increased level of TNF-α induced by EcN_αHL_-UCNPs (NIR+) was observed (Figure S14). Taken together, it was speculated that EcN_αHL_-UCNPs (NIR+) may induce thrombogenesis in tumors to a certain extent, which could transform NIR lasers into thermal energy efficiently 31.

**Figure 5:**
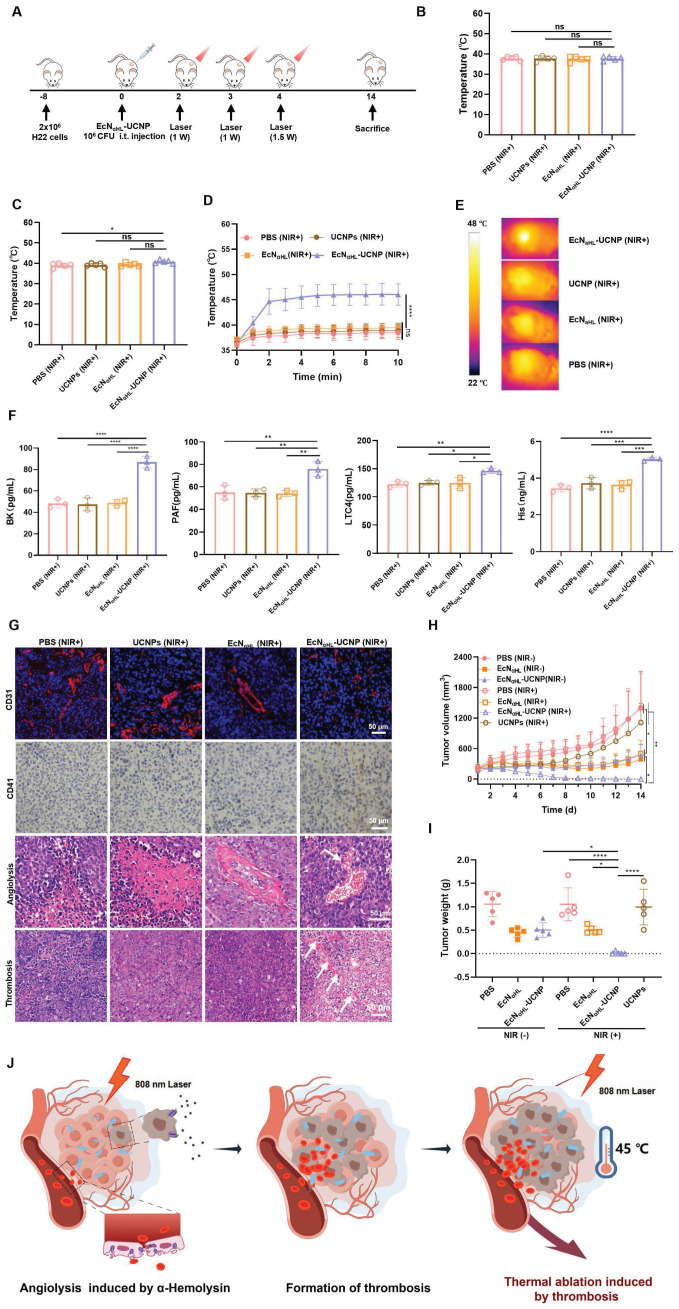
** Optogenetics-based photothermal therapeutic effects evaluation of EcN_αHL_-UCNPs (NIR+) on the subcutaneous H22 tumor model. (A)** Experimental design for evaluating the effects of optogenetics-based photothermal therapy on subcutaneous H22 tumors. Tumor cells were inoculated on day -8 and 1×10^8^ CFU EcN_αHL_ or EcN_αHL_-UCNPs in 100 μL PBS were intratumorally injected on day 0 when the tumor volume reached 100-150 mm^3^. Tumors were irradiated with an NIR laser (808 nm, 1 W cm^-2^, 10 min) on day 2 and 3, with an extra illumination on day 4, (1.5W cm^-2^, 10min) respectively. The temperature of tumor sites in mice injected with bacteria at the doses of 1×10^8^ CFU under 808-nm laser irradiation (1 W cm^-2^, 10 min) for the first time **(B)** and the second time **(C)**. Temperature curves of tumors with different treatments**(D)**, and **(E)** representative IR thermal images of the mice injected under 808 nm laser irradiation (1.5 W cm^-2^, 10 min) for the third time. **(F)** The levels of inflammatory factors including bradykinin (BK), platelet-activating factor (PAF), leukotriene C4 (LTC4), and histamine (His) in sera measured by ELISA kits. **(G)** CD31 staining of tumor vessels (blue color: DAPI staining; red color: CD31), CD41 staining of activated platelets for detecting thrombosis, and hematoxylin and eosin (H&E) staining of tumor tissues, respectively. **(H)** H22 tumor volume and **(I)** tumor weight. **(J)** Schematic illustration of antitumor effects of EcN_αHL_-UCNPs via NIR optogenetical releasing αHL and thrombosis formed subsequently for synergistic photothermal therapy. (*p < 0.05, **p < 0.01, ***p < 0.001, and ****p < 0.0001, ns: no significant difference).

The scheme of photothermal therapy of EcN_αHL_-UCNPs (NIR+) was shown in Figure 5A. When tumor volume reached 100-150 mm^3^, EcN_αHL_-UCNPs(NIR+) at 1×10^8^ CFU were injected intratumorally one time. At 24 h and 48 h after injection of EcN_αHL_-UCNPs (NIR+), the tumors were irradiated by an 808 nm laser at the power density of 1 W cm^-2^ for 10 min to induce the expression of αHL. Tumor temperature and size were measured every day. As recorded by an IR camera, the temperature on bacteria-injected tumors were 38 °C and 40.78 °C, respectively (Figure 5B-C). After being irradiated by an 808 nm laser at the power density of 1.5 W cm^-2^ at 72 h for 10 min, the temperature in EcN_αHL_-UCNPs (NIR+) group increased rapidly to 45 °C, whereas no significant heating effects were found in the PBS, UCNPs (NIR+) and EcN_αHL_ (NIR+) groups (Figure 5D-E). Simultaneously, compared to other control groups, obviously darkened color, and blood clots on tumors in EcN_αHL_-UCNPs (NIR+) group were observed (Figure S15).

Vasodilator inflammatory factors induce vessel-wall injury and the extravasation of blood from the circulation rapidly, and recruit circulating platelets to the site of injury to develop thrombus 18. Vasodilator inflammatory factors such as platelet-activating factor (PAF), leukotriene C4 (LTC4), histamine (His), and bradykinin (BK) in the sera, were measured and increased significantly in EcN_αHL_-UCNPs (NIR+) group compared to other groups (Figure 5F). In addition, CD31 and CD41, typical markers of vascular endothelial cells and platelets, respectively 32, were used for staining tumor vessels and platelets. We found that blood vessels in the EcN_αHL_-UCNPs (NIR+) group were significantly disrupted and platelet aggregation increased compared with the control group. Compared with normal tumor tissue, significant angiolysis and obvious thrombosis could be observed in EcN_αHL_-UCNPs (NIR+) group (Figure 5G).

*In situ* tumor thrombosis possesses performance of photothermal conversion and was utilized as an endogenous photothermal agent to cause tumor heat ablation. We found the tumors of four mice out of five completely disappeared and the tumor inhibition rate achieved to 96.63% in EcN_αHL_-UCNPs (NIR+) group (Figure 5H-I). Tumor growth was suppressed at some extent in EcN (NIR-) group, EcN (NIR+) group and EcN_αHL_-UCNPs (NIR-) group, which may be attributed to poor immunogenicity of EcN. Body weight after injection was decreased on day 1, whereas increased to normal afterwards (Figure S16).

### Immuno-therapeutic effects of NIR light-activatable EcN_αHL_-UCNPs on subcutaneous 4T1 tumor-bearing mice

**Figure 6:**
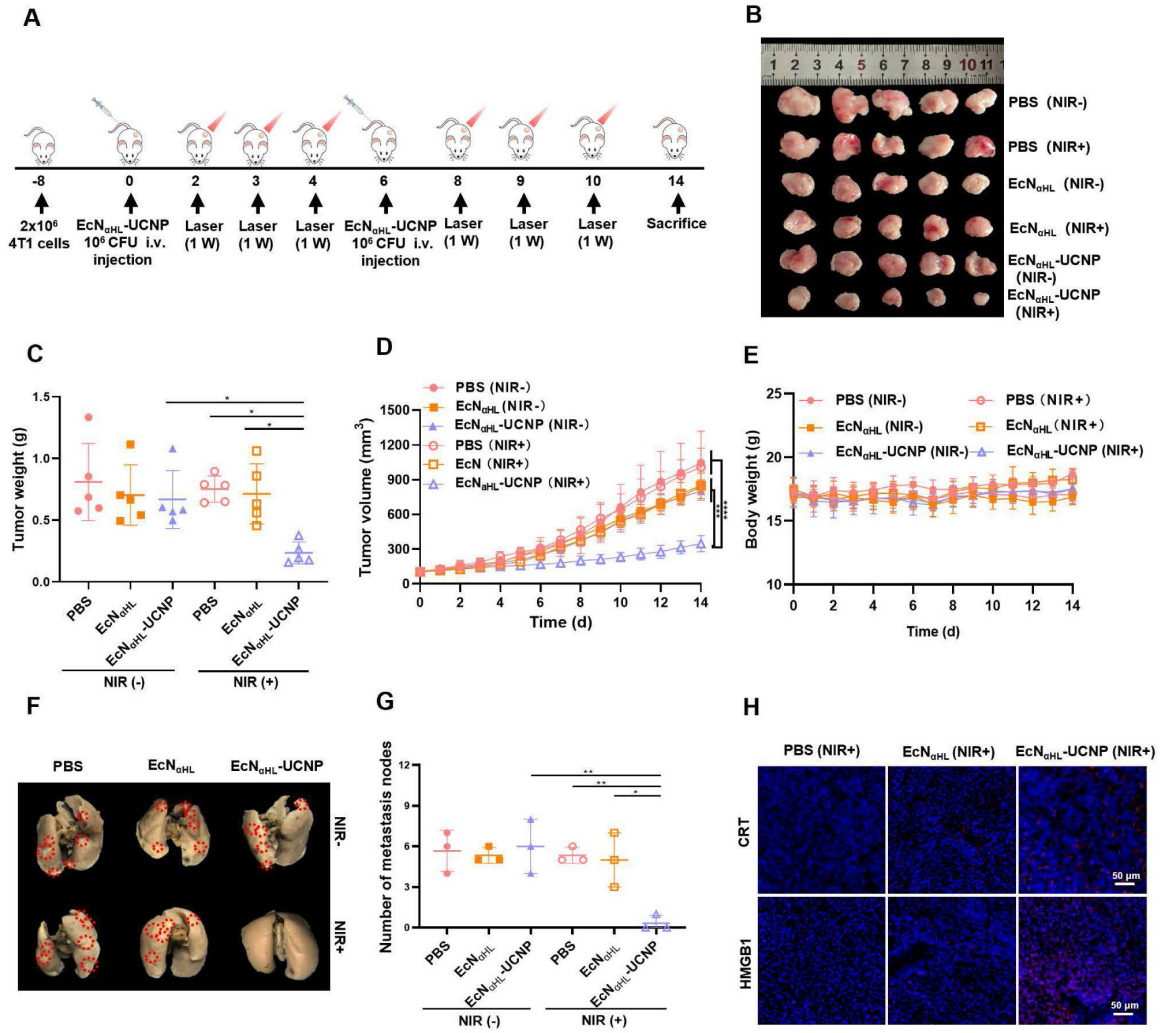
***In vivo* antitumor effects of EcN_αHL_-UCNPs on subcutaneous 4T1 tumor-bearing mice. (A)** Experimental design for evaluating the effects of EcN_αHL_-UCNPs on subcutaneous tumor-bearing mice. Tumor cells were inoculated on day -8 and 1×10^6^ CFU EcN_αHL_ or EcN_αHL_-UCNPs in 100 μL PBS were intravenously injected on day 0 and 6 respectively when the tumor volume reached 100-150 mm^3^. Tumors were irradiated with an NIR laser (808 nm, 1 W cm^-2^, 10 min) on day 2, 3, 4, 8, 9 and 10, respectively. **(B)** 4T1 tumor images and **(C)** tumor weight on day 14. **(D)** 4T1 tumor volume with different treatments. **(E)** Body weight of 4T1 tumor-bearing mice with different treatments. **(F)** Representative images of lung metastatic nodules after different treatments. **(G)** Number of lung metastases was counted as white nodules on the lung surface. **(H)** The immunofluorescence images of CRT (red), HMGB1 (red) of tumors in different groups. Scale bar: 50 μm. (*p < 0.05, **p < 0.01, ***p < 0.001, and ****p < 0.0001, ns: no significant difference).

Based on the results of EcN_αHL_-UCNPs (NIR+) on relative “hot” H22 tumors, we next assessed the therapeutic efficacy of EcN_αHL_-UCNPs on the relative “cold” 4T1 solid tumor (Figure 6A). EcN_αHL_-UCNPs without NIR light irradiation, PBS and EcN_αHL_ with or without NIR light irradiation were employed as controls. Tumor image, tumor volume, tumor weight, and body weight after treatments were shown in Figure 6B-E, respectively. EcN_αHL_ had a modest inhibitory effect on the growth of 4T1 tumor, whereas EcN_αHL_-UCNPs (NIR+) exhibited significant inhibition in tumor growth than other control groups. The tumor inhibition rate in the EcN_αHL_-UCNP (NIR+) group achieved 65.46%. Although the suppression rate of 4T1 tumors was lower than that of H22 tumors, EcN_αHL_-UCNP (NIR+) suppressed the lung metastasis of 4T1 tumor *in vivo* (Figure 6F and G). Immunofluorescence images of CRT and HMGB1 showed that EcN_αHL_-UCNPs (NIR+) could increase the cell surface expression of CRT and the extracellular secretion of HMGB-1 in tumor tissues (Figure 6H).

**Figure 7:**
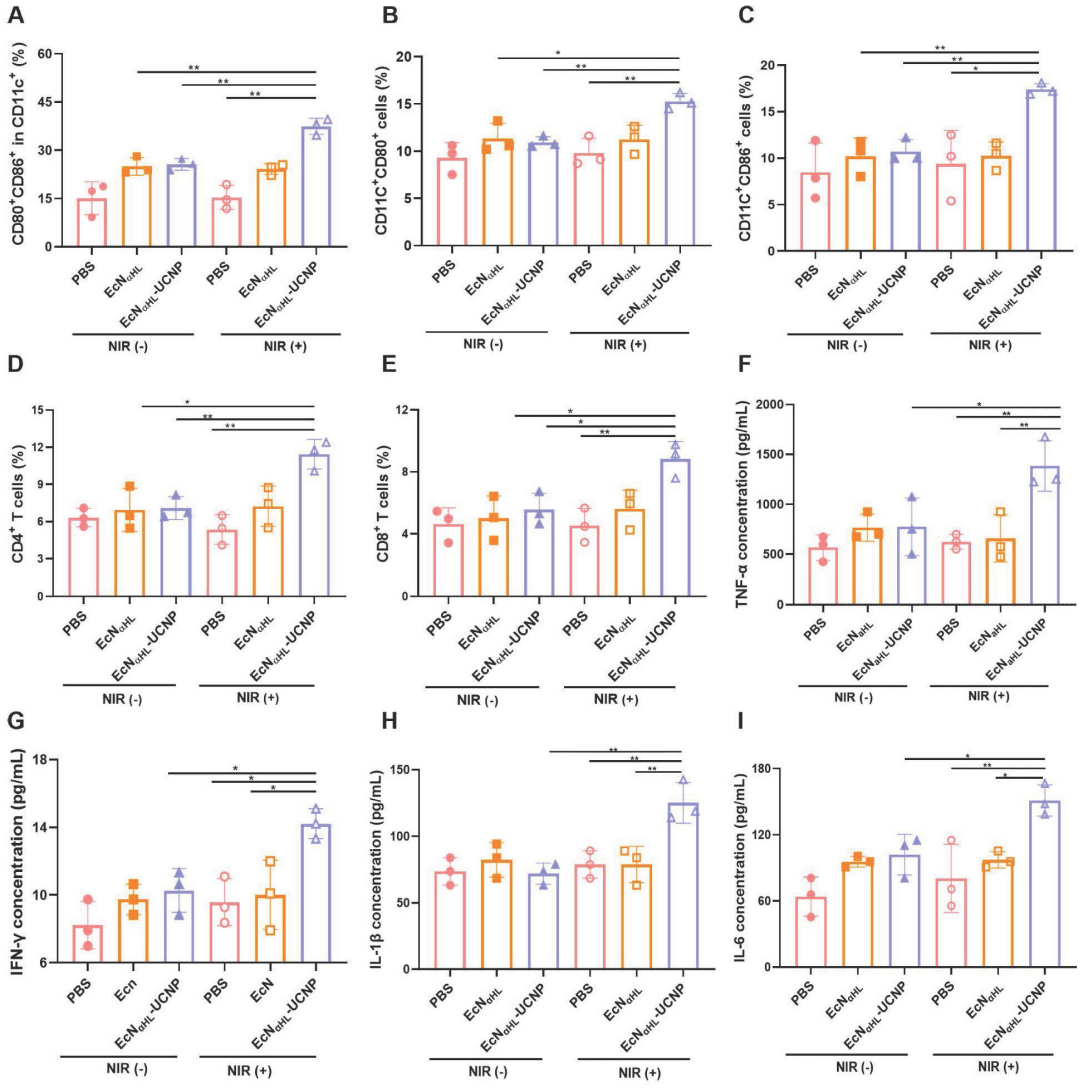
***In vivo* antitumor immune responses induced by EcN_αHL_-UCNPs on subcutaneous 4T1 tumor-bearing mice. (A)** Percentages of CD80^+^ CD86^+^ cells in CD11c^+^ cells in draining lymph nodes. **(B-E)** Percentages of CD11c^+^CD80^+^ cells, CD11c^+^CD86^+^ cells, CD3^+^CD4^+^ T cells and CD3^+^CD8^+^ T cells in tumors. **(F-I)** The concentrations of TNF-α, INF-γ, IL-1β and IL-6 in sera measured by ELISA kits. (*p < 0.05, **p < 0.01, ns: no significant difference).

The immune responses were comprehensively evaluated after treatments with EcN_αHL_-UCNPs (NIR+) in 4T1 tumors. Lymph nodes were harvested on day 3 after last irradiation for analyzing dendritic cells (DCs). As shown in Figure 7A and Figure S17A, CD80^+^ CD86^+^ DCs in lymph nodes in the EcN_αHL_-UCNPs (NIR+) group were 1.46-fold and 1.50-fold higher than that in both EcN_αHL_-UCNPs and EcN_αHL_ without NIR light groups, and 2.45-fold higher than that in the PBS with NIR light group, respectively, which indicated that the expression of αHL induced by NIR could effectively elicit DCs maturation.

The CD80^+^ DCs in tumor tissues in the EcN_αHL_-UCNPs (NIR+) group were 1.40-fold and 1.35-fold higher than that in EcN_αHL_-UCNPs (NIR-) and EcN_αHL_ (NIR-) groups, respectively (Figure 7B). The CD86^+^ DCs in tumor tissues in the EcN_αHL_-UCNPs (NIR+) group were 1.62-fold and 1.70-fold higher than that in both EcN_αHL_-UCNPs (NIR-)and EcN_αHL_ (NIR-) groups (Figure 7C). Moreover, the proportions of both CD4^+^ T and CD8^+^ T cells in the tumor tissues in the EcN_αHL_-UCNPs (NIR+) group were significantly higher than that in other groups (Figure 7D-E and Figure S17B), indicating that EcN_αHL_-UCNPs (NIR+) could promote the infiltration of CD8^+^ T cells into tumor tissues.

Proinflammatory factors play a key role in tumor suppression 33. Therefore, the levels of TNF-α, IFN-γ, IL-1β and IL-6 were analyzed using ELISA kits, and the results showed that EcN_αHL_-UCNPs (NIR+) treatment significantly increased the serum levels of TNF-α, IFN-γ, IL-1β and IL-6 (Figure 7F-I). The level of TNF-α in the EcN_αHL_-UCNPs (NIR+) group was 2.21-fold higher than that in the PBS (NIR+) group and EcN_αHL_(NIR+) group. The level of IFN-γ after different treatments was similar to TNF-α. The level of IL-6 in the EcN_αHL_-UCNPs (NIR+) group was 2.36-fold than that in the PBS group, 1.55-fold than that in the EcN_αHL_ (NIR+) group and 1.48-fold than that in the EcN_αHL_-UCNPs (NIR-) group. The level of IL-1β in EcN_αHL_-UCNPs (NIR+) group was 1.59-fold than that in the EcN_αHL_ (NIR+) group and 1.74-fold than that in the EcN_αHL_-UCNPs (NIR-) group.

### Blood biochemical analysis

Systemic safety of intravenous injection of EcN_αHL_-UCNPs (NIR+) was measured by biochemical analysis (Figure S18). At the end of the experiment, sera were collected for analyzing the levels of aspartate aminotransferase (AST) and alanine aminotransferase (ALT) for liver function, the levels of blood urea nitrogen (BUN) and creatinine (CREA) for kidney function, the level of creatine kinase (CK) for heart function in mice, and the level of lactic dehydrogenase (LDH) for liver, kidney, and heart function. All these markers were in the normal range. Therefore, EcN_αHL_-UCNPs (NIR+) has a good biocompatibility *in vivo.*

## Conclusion

In this study, α-hemolysin was designed to express under blue light generated by lanthanide-doped upconversion nanoparticles (UCNPs) upon NIR irradiation in Escherichia coli Nissle 1917 (EcN). The engineered EcN colonize in tumors and express αHL to disrupt tumor cells and endothelium, resulting in thrombosis formation *in situ* within the tumor. Thrombosis presents a photothermal conversion effect due to its NIR absorption. Thrombosis generated within the tumor was utilized as endogenous *in situ* photothermal agents inducing photothermal ablation of tumors. Thrombosis induced by optogenetics was used for *in situ* PTT has overcome most of the conventional photothermal agents that exhibit disappointed tumor targeting ability, showing a new strategy for cascade tumor therapy.

## Experimental section

### Bacteria and plasmid

The E. coli Nissle 1917(EcN) strain maintained in our lab was used as a host for gene manipulation. The recombinant plasmid pET20b-EL222-pelB-αHL-mCherry harboring the genes coding for blue light-responsive EL222 sensor protein EL222, a reporter mCherry, and αHL (Figure S1), was constructed by Wuhan Miaoling Biotechnology Co. Ltd. The pelB signal sequence within the plasmid pET20b-EL222-pelB-αHL-mCherry can induce the secretion of αHL-mCherry infusion protein. The expression of the recombinant genes was controlled by the T7 promoter. The recombinant plasmid constructed was subjected to DNA sequencing and transformed to EcN by electroporation (2.5 kV, 25 µF, 200 Ω, 5 ms) for further functional verification. Plasmid-transfected EcN (EcN_αHL_) is aerobically cultured in Luria-Bertani (LB) agar plates or LB medium supplemented with antibiotics (100 μg mL^-1^ ampicillin) to maintain the plasmid. The engineered bacteria were cultured in a shaking incubator (180 rpm) at 37 °C. Detailed plasmid is shown in Figure S1.

### Synthesis and surface modification of NaYF4: Yb, Tm@NaYbF4, Nd@NaGdF4 (UCNPs)

The synthesis and modification of UCNPs used in this study has been reported previously 24.

### Preparation of EcN_αHL_-UCNPs

UCNPs (40 mg), EDC (27 mg) and sulfo-NHS (14 mg) were added into the MES buffer (10 mL, 0.1 M, pH 6.0), and then stirred at room temperature for 4 h. After washing with PBS for 3 times, 10^9^ CFU EcN_αHL_ in PBS were added into the deposition, and stirred at room temperature for 12 h. EcN_αHL_-UCNPs were obtained after removing the remaining UCNPs by centrifugation (8000 rpm, 10 min). EcN_αHL_-UCNPs were resuspended in PBS or LB culture medium.

### Characterization of EcN_αHL_, UCNPs and EcN_αHL_-UCNPs

The emission spectra of UCNPs, EcN_αHL_ and EcN_αHL_-UCNPs were measured by time-resolved fluorescence spectrometer (FluoroMax+, Japan) with an additional 808nm laser. Their phenotypes were determined by HT7700 TEM (Hitachi, Japan) at a voltage of 100 kV, and their size and zeta potential were tested by Nano Zetasizer ZS90 (Malvern, UK).

### Counting EcN

Equal count of EcN, EcN_αHL_ and EcN_αHL_-UCNP were inoculated into LB liquid medium at a 1:100 volume ratio for shaking culture, respectively. The OD600 values were measured by UV spectrophotometer (TU-1901 UV-vis) at different culture points (0 h, 1 h, 2 h, 3 h, 4 h, 5 h, 6 h, 7 h, 8 h, 9 h, and 10 h). The equal solution containing EcN_αHL_ or EcN_αHL_-UCNP was coated onto LB solid medium to detect colony formation.

### Induced expression analysis of EcN_αHL_ and EcN_αHL_-UCNPs *in vitro*

To detect the expression of αHL, 5 mL (10^9^ CFU mL^-1^) of EcN_αHL_ and EcN_αHL_-UCNPs were respectively added into LB culture medium with Amp (100 µg mL^-1^), and then 1 mL was taken out and added into an EP tube. The EP tube was shaken at 180 rpm (37 °C), and illuminated with 475 nm blue light or NIR (1W cm^-2^) respectively. Fluorescence intensity of mCherry was measured by fluorescence microscope (Olympus, Japan) and IVIS Lumina XR (Caliper Life Sciences, American). After NIR irradiation for 30 min, 150 μL of EcN_αHL_-UCNPs solution were taken out, and fluorescence signal was detected at 0 h, 0.5 h, 1 h, and 2 h by flow cytometry to investigate the expression level. Furthermore, different power intensity (0, 0.5, 1.0 and 1.5 W cm^-2^) were used to explore the fluorescence intensity of mCherry.

The expression of αHL was analyzed by Western blot. Anti-mCherry antibody (Proteintech, China) at 1:1000 dilution ratio was added into the lysis of blue light-induced EcN_αHL,_ NIR-induced EcN_αHL_-UCNPs and their supernatants, at 4 °C overnight, and then incubated with horseradish peroxidase conjugated secondary antibody for 1 h at room temperature. Chemiluminescence imaging system (ChemiDoc XRS, Bio-Rad, USA) was used to analyze the electrophoretic bands.

### Cell culture and cytotoxicity of EcN_αHL_-UCNPs *in vitro*

H22 and 4T1 cells were cultured in RPMI 1640 medium with 10% fetal bovine serum (containing 100 U mL^-1^ penicillin and 100 μg mL^-1^ streptomycin) at 37 °C in 5% CO_2_ incubator. BMDC were obtained from bone marrow cells induced by GM-CSF (10 ng mL^-1^) and IL-4 (10 ng mL^-1^) for 7 days.

Hemolysis of EcN_αHL_ after irradiation was analyzed by blood agar plates. Wild type EcN, EcN_αHL_ and EcN_αHL_-UCNP were coated on the blood agar plates overnight, subsequently irradiated by blue light or NIR for 30 min, and then cultured for 5 h at 37 °C. Images were taken to investigate the hemolysis.

EcN_αHL_-UCNP (1×10^6^ CFU) were added into a 96 well plate with 1×10^4^ cells/well to evaluate the cytotoxicity of αHL secreted by the irradiated EcN_αHL_-UCNP. LDH was detected by a kit according to the operating manual.

4T1 cell viability was detected by the MTT method. EcN_αHL_ and EcN_αHL_-UCNPs were irradiated by blue light or NIR respectively, and then the supernatants were collected for incubation with 2×10^5^ 4T1 cells for 24 h. After washing with PBS 2 times, 80 μL of MTT were added and co-incubation for 4 h. The absorbance was measured at 570 nm. The H22 tumor cell viability was determined by a CCK8 kit.

The supernatants collected from irradiated EcN_αHL_- UCNPs were incubated with 2×10^5^ 4T1 cells for 24 h. Subsequently, flow cytometry was used to analyze apoptosis.

### Detection of immunogenic cell death *in vitro*

The supernatants harvested from irradiated EcN_αHL_- UCNPs were incubated with 2×10^5^ 4T1 cells for 24 h. Cells were collected after washing with PBS. Anti-CRT antibody was added after blocking with 1% BSA for 0.5 h. One hour later, FITC conjugated secondary antibody was added. Flow cytometry was used to analyze the level of CRT. At the same time, HMGB1 was detected using an ELISA kit according to the manual.

### The BMDC maturation analysis *in vitro*

Mice bone marrow monocytes were extracted and stimulated to differentiate into the immature BMDCs by GM-CSF and IL-4 *in vitro*. The EcN_αHL_ supernatants collected from irradiated EcN_αHL_-UCNPs was incubated with 4T1 cells, and then 4T1 cell supernatants were added into the immature BMDCs. Undergoing co-incubation for 24 h, BMDCs were stained by anti-CD80-APC, anti-CD11c-FITC and anti-CD86-APC for 30 min. After washing with PBS, flow cytometry was used to analyze the stained cells.

### Animals

BALB/c mice (6~8 weeks old, 17~20 g) were purchased from Liaoning Changsheng biotechnology co., Ltd. (Liaoning, China). Mice were bred under a constant environmental condition (22 ± 2 °C; 55 ± 5% relative humidity, and a 12-h light-dark cycle). 4T1 or H22 cells (2×10^6^) were injected subcutaneously into the flank of BALB/c mice to construct a subcutaneous 4T1 or H22 tumor-bearing mouse model. Animal studies were approved by the Institutional Animal Care and Use Committee of Huazhong University of Science and Technology (IACUC Number: 3662).

### *In vivo* biodistribution analysis of EcN_αHL_-UCNPs

IR780-stained EcN_αHL_-UCNPs were injected via i.v. at 1×10^6^ CFU into a 4T1 tumor-bearing BALB/c mouse to investigate the distribution of EcN_αHL_-UCNPs *in vivo*. At different time points (0 h, 0.5 h, 1 h, 2 h, 4 h, 8 h, 12 h, 24 h, 48 h, and 72 h) after injection, fluorescence images were taken by IVIS Lumina XR. Main organs (hearts, livers, spleens, lungs, and kidneys, at 12 h, 48 h and 7 d), and tumors (at 2 h, 12 h, 24 h, 48 h, 72 h and 7 d) were respectively collected after injection of EcN_αHL_-UCNPs suspensions. EcN_αHL_-UCNPs in these tissues were quantified by coating LB plates with the tissue homogenates overnight. Moreover, Yb, Nd and Y of UCNPs in different tissues were analyzed by ICP-OES after injection at 72 h.

### Evaluation of antitumor effects *in vivo*

For evaluating the effects of ECN_αHL_-UCNP (NIR+) on H22 tumor-bearing mice, PBS (100 μL), EcN_αHL_ (10^6^ CFU in 100 μL PBS), or EcN_αHL_ -UCNPs (10^6^ CFU in 100 μL PBS) were subcutaneously injected 2 times on day 0 and 6 when tumor volume of H22 tumor reached about 100-150 mm^3^. 808 nm NIR light illumination (1 W cm^-2^, 10 min) was performed on day 2, 3, 4, 8, 9, and 10, respectively. For further evaluating the photothermic therapy effect of ECN_αHL_-UCNP (NIR+), 7 groups were divided: PBS (NIR-), PBS (NIR+), UCNPs (NIR+), ECN_αHL_ (NIR-), ECN_αHL_ (NIR+), ECN_αHL_-UCNP (NIR-), and ECN_αHL_-UCNP (NIR+). When tumor volume reached about 100-150 mm^3^, 1×10^8^ CFU bacteria were injected into the tumor on day 1. NIR light illumination (808 nm, 1 W cm^-2^, 10 min) was performed on day 2 and 3. In addition, NIR light illumination at 1.5 W cm^-2^ for 10 min was performed on day 4 (Unscabbed state). Infrared thermal images were photographed. The body weight and tumor size were measured every day. Tumor images were photographed at the end of experiments.

4T1 tumor-bearing mice were used to determine the antitumor efficacy. Six groups were divided as follows: PBS (NIR-), PBS (NIR+), EcN_αHL_ (NIR-), EcN_αHL_ (NIR+), EcN_αHL_-UCNP (NIR-), and EcN_αHL_-UCNP (NIR+). When tumor volume of 4T1 tumor reached about 100-150 mm^3^, the mice were injected via i.v. with PBS (100 μL), EcN_αHL_ (10^6^ CFU in 100 μL PBS), or EcN_αHL_ -UCNPs (10^6^CFU in 100 μL PBS) 2 times on day 0 and 6. 808 nm NIR light illumination (1 W cm^-2^, 10 min) was performed on day 2, 3, 4, 8, 9, and 10, respectively. The body weight and tumor size were measured every day. On day 7, lymph nodes from different groups were collected for analysis of DC maturation by flow cytometry. On day 14, tumors were collected, weighted, photographed, and analyzed by flow cytometry. Major organs (heart, liver, spleen, lung, and kidney) were resected for pathological analysis. Complete lung tissues from different groups were fixed in Bouin's solution (Solarbio, Beijing, China) for 12 h at room temperature, and metastatic nodules were counted.

### Flow cytometry analysis

The lymph nodes tissues were mashed to obtain single-cell suspensions through a 40 µm cell strainer for DCs analysis, and then stained by fluorescently labeled anti-CD11c, anti-CD86 and anti-CD80 (BioLegend, USA). The pieces of the tumor tissues were digested with 0.8 mg mL^-1^ collagenase I for 40 min at 37 °C, and then washed with PBS. Subsequently, the suspensions were filtrated by a syringe plunger on a 40 µm cell strainer to obtain single-cell suspensions. For T cells analysis, the cells were stained by fluorescently labeled anti-CD45, anti-CD3ε, anti-CD4, and anti-CD8a (BioLegend, USA). Cytoflexs flow cytometry was used to analyze the stained cells and the CytExpert software (Beckman, USA) was used to process the data.

### Tissue staining

At the end of experiments, major organs and tumor tissues were fixed in 4% paraformaldehyde for 24 h at room temperature. Pathological changes of heart, liver, spleen, lung, and kidney tissues were detected by H&E staining. Ki67 (a proliferation marker) staining and TUNEL (for detecting apoptotic cells) were carried out in Wuhan Sevicebio Technology Co., Ltd. (Wuhan, China). The images were captured with a light microscope (NIKON, Japan) and a fluorescence microscope.

### Analysis of inflammatory factors in sera and blood biochemistry

At the end of the experiments, blood samples from mice were centrifuged (3000 rpm, 10 min) to obtain sera. After dilution with PBS, part of sera was used to measure the levels of TNF-α, IFN-γ, IL-6 and IL-1β by the kits according to the manual. The other part was used to determine the levels of CREA, AST, ALT, BUN, and CK using an automatic biochemical analyzer (Mindary, China) in the Hospital of Huazhong University of Science and Technology.

### Detection of vasodilator inflammatory factors

The serum levels of vasodilator inflammatory factors, including BK, LTC4, His, and PAF were detected using ELISA kits. Briefly, the whole blood of H22 tumor-bearing mice was collected at day 5 after the intratumoral injection of bacteria. After standing for 20 min, the whole blood samples were centrifuged at 2000 rpm for 20 min to collect the supernatant. The relevant tests were performed according to the manual.

### Thrombosis analysis

The mice were randomly divided into four groups and treated with PBS (NIR+), UCNPs (NIR+), ECN_αHL_ (NIR+) (10^8^ CFU per mouse), or ECN_αHL_-UCNP (NIR+) (10^8^ CFU per mouse). 808 nm NIR light illumination (1.0 W cm^-2^, 10 min) was performed on day 1, 2, and day 3 (1.5 W cm^-2^, 10 min), respectively. Five days post-injection, mouse tumor images were taken. The tumor tissues were sliced and stained with an anti-CD31 antibody for analysis of blood vessels and an anti-CD41 antibody for analysis of activated platelets.

### Statistical analysis

Statistical analysis was performed using GraphPad Prism 8.0 software, and the results were presented as mean (s. e. m). Two-tailed Student's T-test or one-way ANOVA followed by Turkey's test was performed. *p*<0.05 was considered to be significantly different. (*p < 0.05, **p < 0.01, ***p < 0.001, and ****p < 0.0001, ns: no significant difference).

## Supplementary Material

Supplementary figures.

## Abbreviations

EcN: Escherichia coli Nissle1917
UCNPs: Upconversion Nanoparticles
DCs: Dendritic cells
αHL: α-Hemolysin
ICD: Immunogenic cell death
CRT: Calreticulin
HMGB1: High mobility group protein B1
NIR: Near infrared light
IFN-γ: Interferon-γ
IL-6: Interleukin-6
TNF-α: Tumor Necrosis Factor-α
IL-1β: Interleukin-1β
PTT: Photothermal therapy
WB: Western Blot
