# Supplementary material for: Bacteria-based cascade in situ near-infrared nano-optogenetically induced photothermal tumor therapy
Source: Theranostics. 2024 Aug 12;14(13):4933–47. doi: 10.7150/thno.98097 (PMC11388082; doi:10.7150/thno.98097)
Supplement: Supplementary file 1 — Supplementary figures. [file thnov14p4933s1.pdf]

Supplementary information

Bacteria-based cascade in situ near-infrared nano-optogenetically induced photothermal tumor therapy

Xiuwen Hu[#], Jiawen Chen[#], Yuzhi Qiu, Sihan Chen, Yidi Liu, Xi Yu, Yunting Liu, Xiangliang Yang*, Yan Zhang*, and Yanhong Zhu*

National Engineering Research Center for Nanomedicine, College of Life Science and Technology, Huazhong University of Science and Technology, 1037 Luoyu Road, Wuhan 430074, P. R. China

* Corresponding authors: Yanhong Zhu, Yan Zhang, Xiangliang Yang, National Engineering Research Center for Nanomedicine, College of Life Science and Technology, Hubei Key Laboratory of Bioinorganic Chemistry and Materia Medica, Huazhong University of Science and Technology. E-mail: yhzhu@hust.edu.cn, yan_zhang@hust.edu.cn, nanomedicine@mail.hust.edu.cn.

# These authors contributed equally to this study.

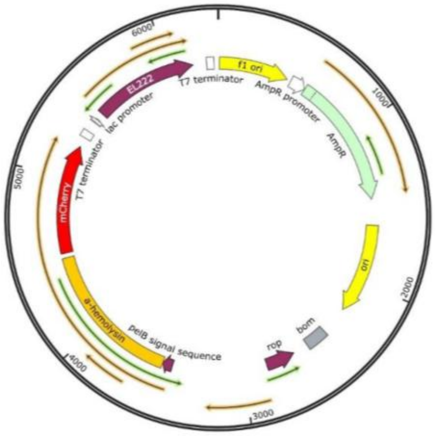

Figure S1. The plasmid structure of pET20b-EL222-αHL-mCherry

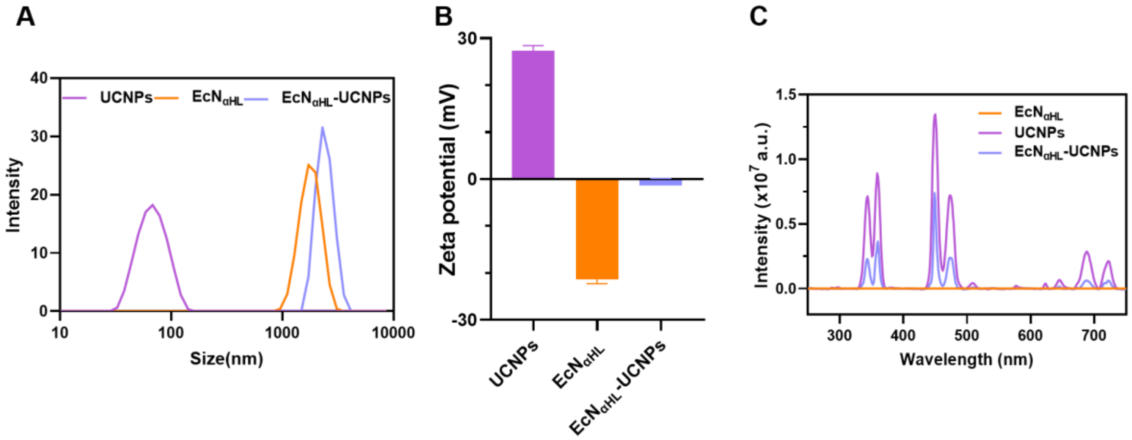

Figure S2. (A) The hydrated particle size of $EcN_{\alpha HL}$, UCNPs and $EcN_{\alpha HL}$-UCNP. (B) Zeta potential of $EcN_{\alpha HL}$, UCNPs and $EcN_{\alpha HL}$-UCNP. (C) The emission spectra of $EcN_{\alpha HL}$, UCNPs and $EcN_{\alpha HL}$-UCNP.

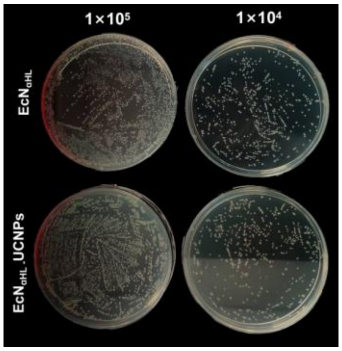

Figure S3. The colony formation and colony morphology of $EcN_{\alpha HL}$ and $EcN_{\alpha HL}$-UCNPs.

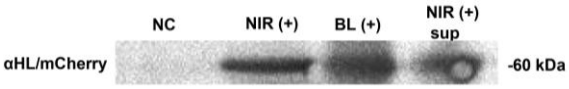

Figure S4. Western blot of αHL-mCherry fusion protein detected by anti-mCherry antibody. NC: $EcN_{\alpha HL}$ without light; NIR+: $EcN_{\alpha HL}$-UCNP irradiated by NIR at 1 W cm^{-2} for 2 h; BL+: $EcN_{\alpha HL}$ irradiated by blue light for 2 h; NIR+Sup: supernantant of NIR+ group.

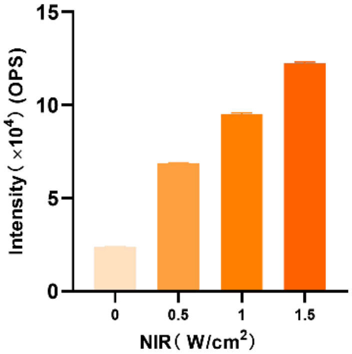

Figure S5. The mCherry fluorescence intensity of bacterial medium of $EcN_{\alpha HL}$-UCNPs after irradiation of NIR with different intensities for 30 min.

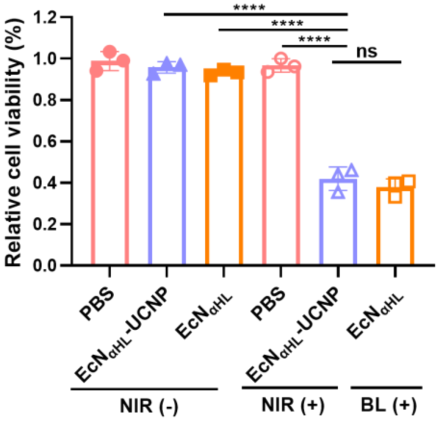

Figure S6. The relative cell viability of H22 cells treated by $EcN_{\alpha HL}$-UCNPs irradiated with or without NIR.

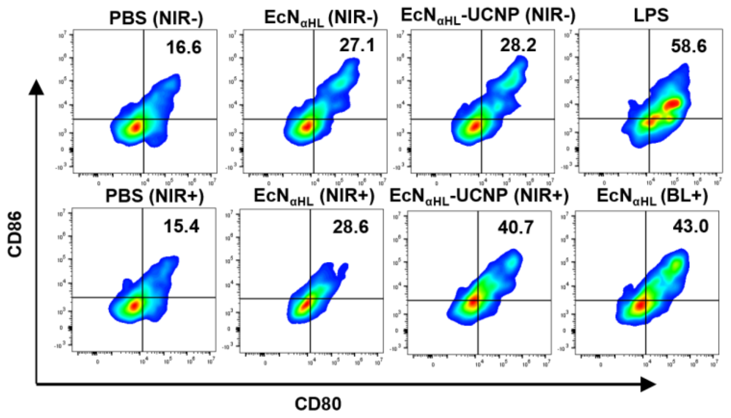

Figure S7. Flow cytometric analysis of mature dendritic cell (DC) ($CD80^+CD86^+$ of $CD11c^+$) in vitro.

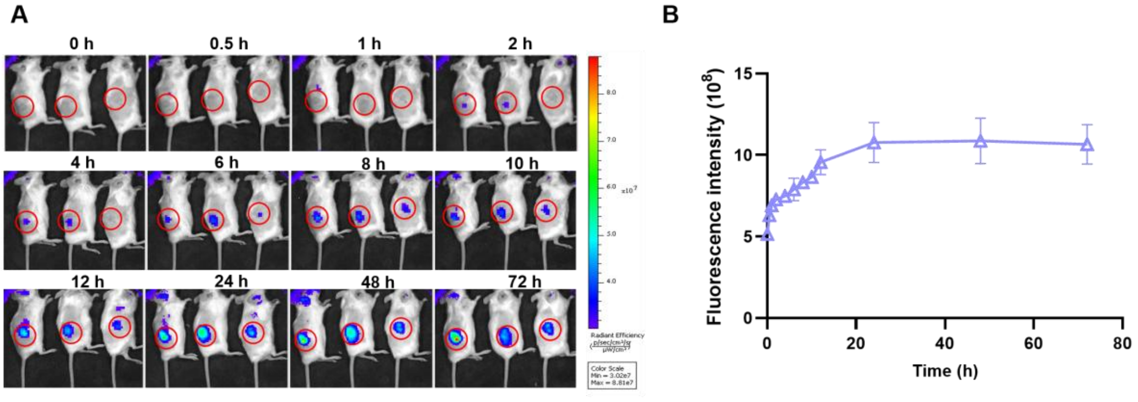

Figure S8. (A) The in vivo fluorescence images of IR-780-labelled $EcN_{\alpha HL}$-UCNPs and average fluorescence intensity in tumors (B).

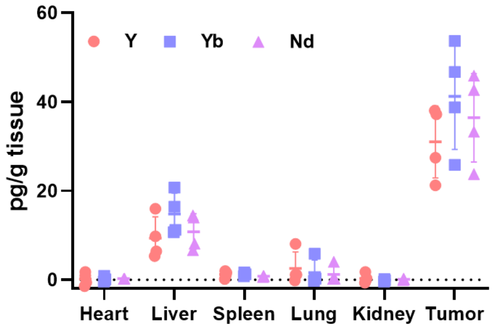

Figure S9. The content of Yb, Nd, and Y in heart, liver, spleen, lung, kidney, and tumor at 96 h after intravenous injection of $EcN_{\alpha HL}$ -UCNPs (1×10^6 CFU per mouse). All values are expressed as mean ± s.e.m. (n=4)

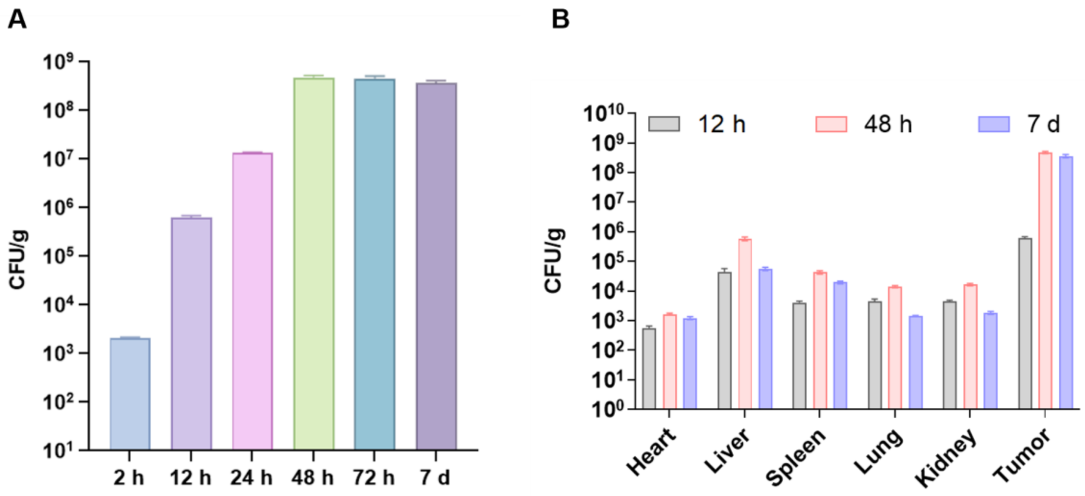

Figure S10. The tumor colonization of $EcN_{\alpha HL}$-UCNPs (A) and the distribution of $EcN_{\alpha HL}$-UCNPs in hearts, livers, spleens, lungs, kidneys and tumors at different time points in 4T1 tumor bearing BALB/c mice after intravenous injection (1×10^6 CFU per mouse). (n=5)

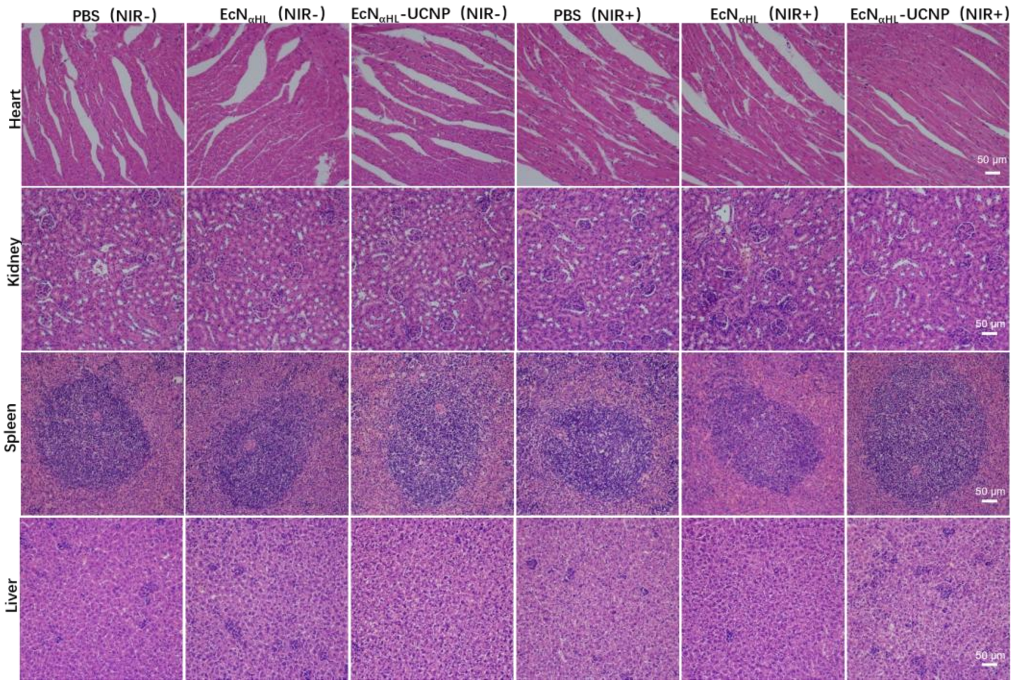

Figure S11. H&E staining images of heart, kidney, spleen, and liver tissues in subcutaneous H22 tumor-bearing mice. Scale bar: 50 μm.

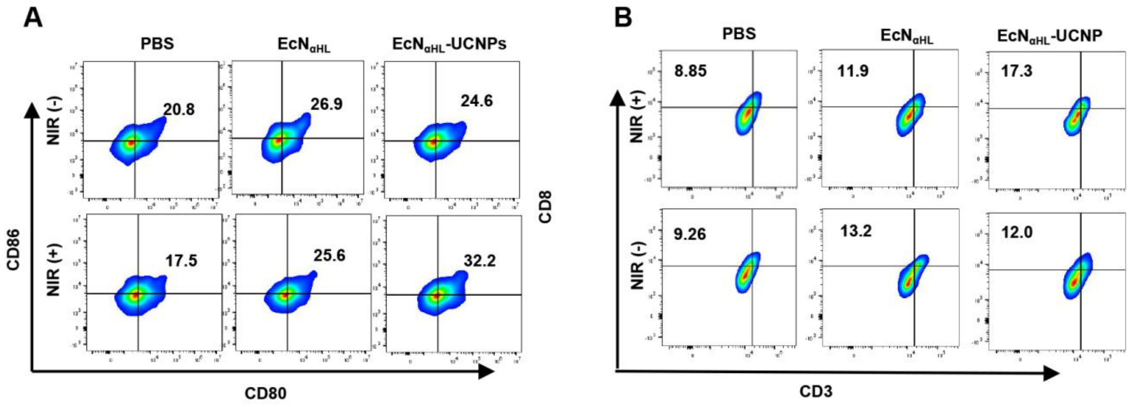

Figure S12. (A) Flow cytometric analysis of mature dendritic cell (DC) ($CD80^+CD86^+$ of $CD11c^+$) in H22 draining lymph nodes. (B) Flow cytometric analysis of $CD3^+CD8^+$ T cells in H22 tumor tissues.

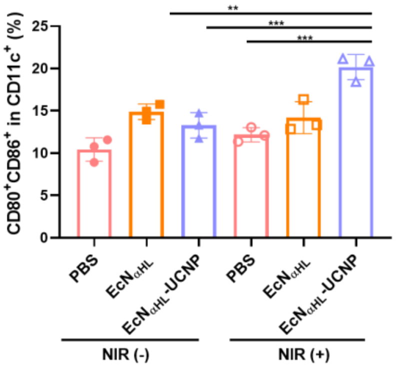

Figure S13. Percentages of $CD80^{+}$ $CD86^{+}$ cells in $CD11c^{+}$ cells in H22 tumor tissues.

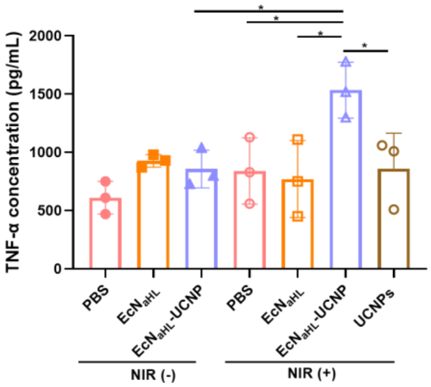

Figure S14. The concentration of TNF-α in sera measured by ELISA kit in H22 tumor-bearing mice.

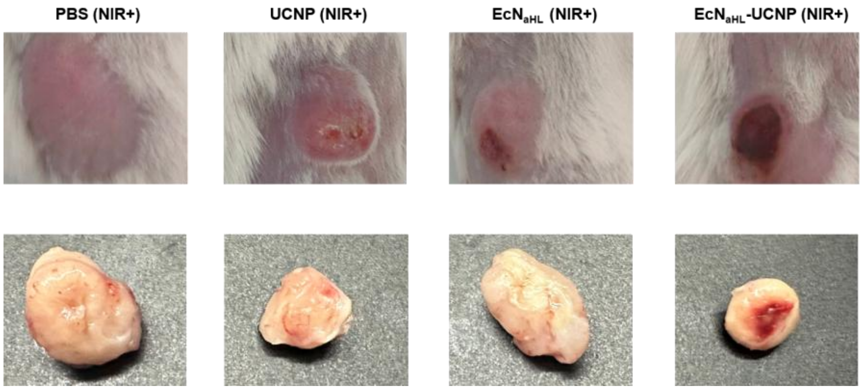

Figure S15. Photographs of tumor-bearing BALB/c mice and tumor tissues after different treatments on day 5.

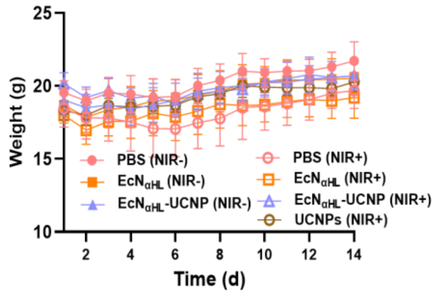

Figure S16. Body weight of H22 tumor-bearing mice with different treatments.

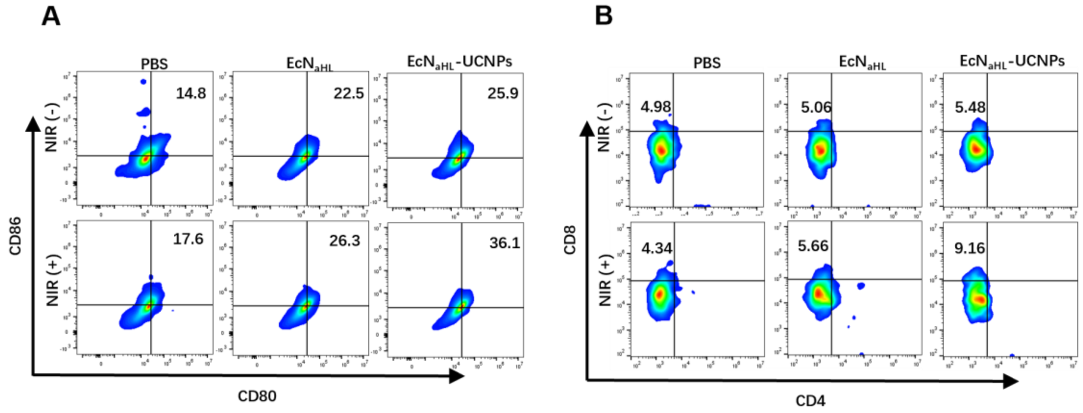

Figure S17. (A)Flow cytometric analysis of mature dendritic cell (DC) ($CD80^+CD86^+$ of $CD11c^+$) in 4T1 draining lymph nodes. (B) Flow cytometric analysis of $CD3^+CD8^+$ T cells in 4T1 tumor tissues.

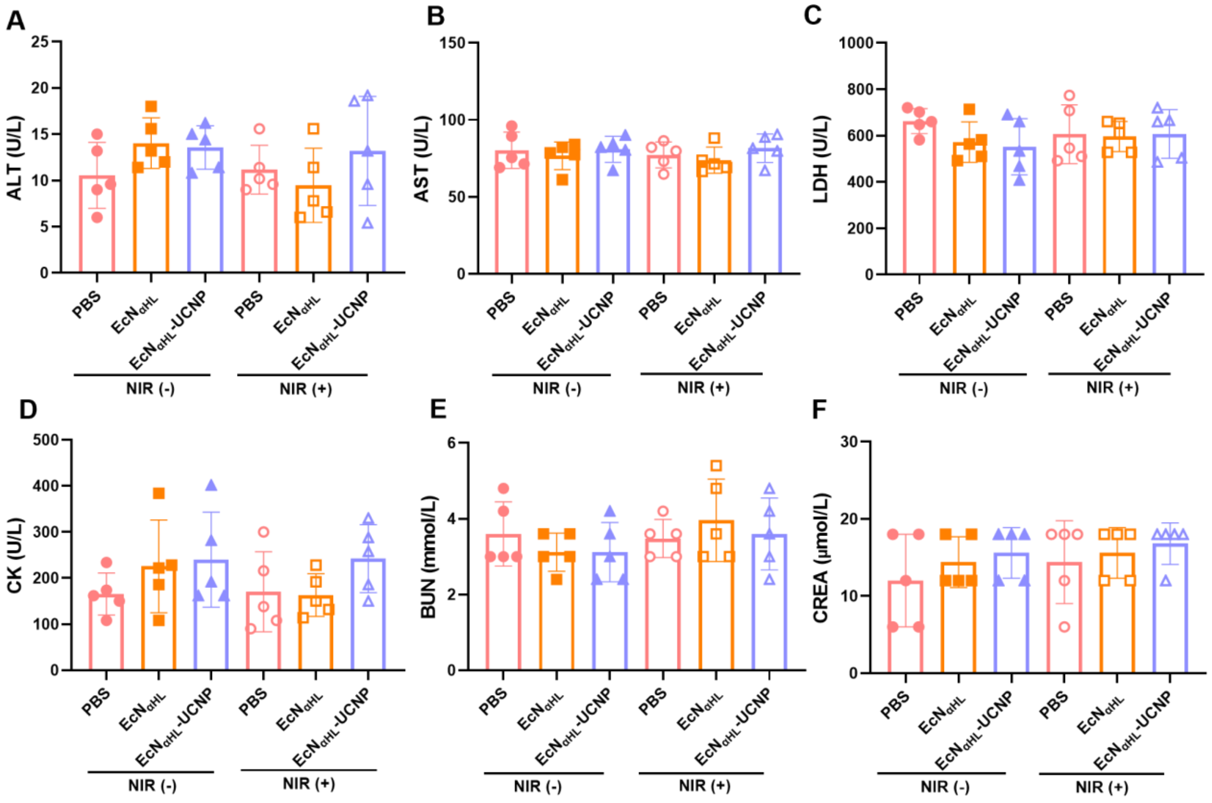

Figure S18. Blood biochemical analysis in 4T1 tumor-bearing mice. The levels of BUN (A), CK (B), LDH (C), CREA (D), AST (E), and ALT (F) in sera of mice after different treatments. All values are expressed as mean ± s.e.m. (n = 5).
